# Graphene and zeolite as adsorbents in bar-micro-solid phase extraction of pharmaceutical compounds of diverse polarities

**DOI:** 10.1039/d1ra01569a

**Published:** 2021-05-04

**Authors:** Maizatul Najwa Jajuli, Grégoire Herzog, Marc Hébrant, Ng Eng Poh, Afidah Abdul Rahim, Bahruddin Saad, M. Hazwan Hussin

**Affiliations:** Department of Chemistry, Faculty of Science and Mathematics, Sultan Idris Education University 35900 Tanjong Malim Perak Malaysia najwa@fsmt.upsi.edu.my; Université de Lorraine, CNRS, LCPME F-54000 Nancy France; School of Chemistry, Universiti Sains Malaysia 11800 Pulau Pinang Malaysia mhh@usm.my; Fundamental and Applied Sciences Department, Universiti Teknologi PETRONAS 32610 Seri Iskandar Perak Darul Rizwan Malaysia

## Abstract

A bar micro-solid phase (bar μ-SPE) extraction method using either graphene or zeolite or their mixtures as an adsorbent, coupled with high-performance liquid chromatography (using a C1 column) was developed for the simultaneous determination of pharmaceutical compounds (metformin (MET), buformin (BUF), phenformin (PHEN) and propranolol (PROP)) of diverse polarity (log *P* from −1.82 to 3.10). Parameters influencing the extraction, such as conditioning solvents, pH of the sample, sample volume, amount of adsorbent, stirring rate, time of extraction, type and volume of desorption solvent and time of desorption were investigated. Under the optimized conditions, the extraction method using graphene (extraction efficiency, % EE, ∼6–15%) resulted in the least amount of extracted drugs. However, the use of zeolite and zeolite/graphene mixtures improves the % EE significantly, *i.e.* 30% for PHEN and 42% for PROP using zeolite; 22% for MET and 18% for BUF using the adsorbent mixture. Under similar conditions, enrichment factors for these drugs range from 11–15. The validated method was performed for the determination of the drugs that were spiked to urine samples. Good recoveries ranging from 72.8 to 116% were achieved.

## Abstract 2

A bar micro-solid phase (bar μ-SPE) extraction method using either graphene or zeolite or their mixtures as an adsorbent, coupled with high-performance liquid chromatography was developed for the determination of pharmaceutical compounds of diverse polarity.

## Graphical abstract

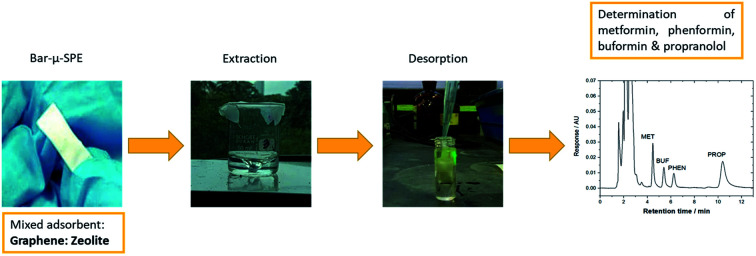

## 1.0 Introduction

The simultaneous determination of compounds with diverse polarities is mandatory for some specialized applications. An example is in doping control where all non-permitted substances, covering a large window of polarities, need to be identified and confirmed. The simultaneous extraction of these compounds shortens the crucial turnaround time as per the World Anti-Doping Agency requirements. Simultaneous extraction of compounds with a diverse chemical structures and wide range of polarities is a challenging task. The relative polarities of compounds can be expressed by the partition coefficient (*P*), defined by the ratio of the analyte's concentration in *n*-octanol relative to water.^1^ This classification is particularly useful for pharmacists to reflect the fundamental properties of the particular compound (*e.g.*, negative log *P* is associated with polar compounds with good aqueous solubility, while positive log *P* is related to non-polar compounds with good lipid solubility, but poor aqueous solubility). Log *P* is also used for assessing bioaccumulation, health, ecological toxicity^2^ and in the development of liquid–liquid extraction (LLE), liquid-phase microextraction and their related techniques^3–6^ including with the aid of ultrasonication.^7–10^

The traditional method of LLE extracts only a polar or non-polar compound. The polarity of the extracting solvent mainly dictates the extraction. Solid-phase extraction (SPE) offers substantial improvements over the LLE, especially in reducing the use of organic solvents.^11,12^ SPE using the non-polar C18 adsorbent (the most widely used adsorbent) generally for the extraction of non-polar compounds.^13^ The extraction of highly polar compounds, such as amines is not suitable using both the LLE and SPE techniques due to the hydrophilic molecules preferred to retain in the aqueous phase.

Basheer *et al.*^14^ introduced a stimulating electromembrane extraction (EME) method for the simultaneous extraction of acidic and basic drugs: betaxolol (p*K*_a_ 9.67, log *P*, 2.54); diclofenac (p*K*_a_ 4.00, log *P*, 4.26); mefenamic acid (p*K*_a_ 3.89, log *P* 5.40). In their well-designed set-up, four sheets of porous polypropylene membrane were heat-sealed at three edges, platinum electrodes were inserted in each side of membrane pocket, and a dc voltage was used to apply an electrical potential between them. The acidic and basic drugs were first extracted into the aqueous phase filled into the side membrane pockets and next transferred into the organic acceptor phase filled into the middle membrane pocket. The organic acceptor phase was finally analyzed using gas chromatography-mass spectrometry (GC-MS). This work inspires the development of other EME techniques aimed at the simultaneous extraction of acid and base compounds of a wide polarity window.^15–18^

The SPE technique, focusing on novel adsorbent materials continues to attract the attention of researchers in the quest for the simultaneous extraction of compounds with different polarity window. The mixed-mode polymeric sorbent (*e.g.*, Oasis MCX cartridge), containing reversed-phase (C18) and cation exchange (sulfonic) functional groups are well established for the extraction of a wide range of compounds. They are used to adsorb both polar and non-polar, neutral and cationic compounds concurrently from the aqueous media *via* mixed-mode robust cation exchange and reversed-phase mechanisms. Three-dimensional honeycomb Mg–Al layered double oxide combined with graphitized carbon black was also used as the SPE adsorbent for the simultaneous determination of 15 pesticide residues in green tea coupled with GC-MS.^19^ Recovery values of 71.1–119.0% were attained for the tested pesticides. In another study, Roy *et al.*^20^ described the synthesis of polymeric adsorbents with tunable surface polarity and their application as SPE for the determination of polar and non-polar chemical warfare agents in non-polar matrices. Through the proper selection of monomer, cross-linker and solvent in the synthesis step, polymeric adsorbents with the required polarity can be obtained. Four types of polymeric sorbent containing two or more monomers (methacrylic acid, divinylbenzene, hydroxyethyl methacrylate, and ethylene glycol dimethacrylate) with different composition were synthesized. Polymer containing methacrylic acid and ethylene glycol dimethacrylate with ratio 1 : 2 was chosen as it has higher polarity imparted by the presence of carboxyl and carbonyl functional groups. It was evident extraction using polymeric sorbent (methacrylic acid and ethylene glycol dimethacrylate, 1 : 2) is more efficient than silica sorbent.^20^

Zhang and Zhou *et al.* tested several SPE cartridges for simultaneous determination of eleven pharmaceutical compounds using SPE technique. Among the tested cartridges, Oasis HLB-SPE cartridge, the copolymer cartridge was found to produce the best recoveries for the targeted analytes.^21^ Zhang and co-workers demonstrated the simultaneous determination of illicit drugs in biological samples using Oasis MCX cartridges to extract twelve illicit drugs such as amphetamine, metamephamine, cathinone, mathecathinone and *N*-methylephedrine.^22^ Domínguez-Romero *et al.* assessed seven different sample treatments, including SPE using polymeric cartridge (PLEXA polymeric cartridges and Oasis HLB) and mixed-mode ion cartridges (Oasis MCX) for large-scale multiclass sports drug testing.^23^ Analysis of 34 diuretics and beta-blockers in urine was done by Marchi *et al.* using Oasis Sorbent Selection Plate (comprising Oasis MCX, Oasis MAX, Oasis WCX and Oasis WAX sorbents and Oasis HLB).^24^

The present study reports the development of an alternative method for the simultaneous extraction of pharmaceutical compounds of diverse polarity based on the bar-micro-solid phase extraction (bar-μ-SPE).^25^ The μ-SPE technique was first introduced by Basheer *et al.*^26^ in 2006 to overcome the shortcomings of the SPE technique. The benefits of using μ-SPE are the lower consumption of solvents, low-cost, simplicity, and ease of operation. The μ-SPE device was prepared by placing a few milligrams of the adsorbent inside a heat-sealed porous membrane. By inserting a metal rod in the membrane, the device was improved to overcome the inefficient mixing of the sample and the adsorbent induced by the device's propensity to float on top of the sample surface. This modified device is known as the bar μ-SPE.^25^ The new device was fully immersed in the sample solution, hence improving the extraction. Key to the success of the bar-μ-SPE extraction is the choice of adsorbents used. Due to the diverse polarities of the drugs used in the present study, the use of a mixture of adsorbents of contrasting surface properties (*e.g.*, polar and non-polar) was rationalized to achieve the objectives. Graphene has recently acquired considerable attention in sample preparation.^27,28^ Graphene is characterized by an ultra-high surface area, double-sided surface area, excellent chemical and thermal stability.^29–31^ Luo *et al.* and Naing *et al.*, had reported the use of graphene as an adsorbent in SPE for phthalate esters in aqueous solution^32^ and polar estrogen in water,^33^ respectively. Meanwhile, Shen *et al.* had reported the extraction of marine toxins in shellfish using the graphene-based pipette tip SPE method.^34^ In contrast, zeolites are open-framework aluminosilicates with well-defined microporous channels. Fernández *et al.* had studied the use of zeolite with iron oxide composite as sorbent for magnetic SPE of benzene, toluene, ethylbenzene and xylenes from water^35^ Zeolite LTL is a one-dimensional 12 membered-ring channel system. Zeolite LTL with different pore sizes (diameter ranging from 200 to 2000 nm) and channels can be synthesized, depending on the experimental conditions. It is effective for the extraction of the mycotoxin ochratoxin A in coffee and cereals.^36^

In this work, we have evaluated the use of zeolite, graphene and their mixtures in bar μ-SPE for the simultaneous extraction of the pharmaceuticals (metformin (MET), buformin (BUF), phenformin (PHEN), and propranolol (PROP)), of diverse polarity (log *P* from −1.82 to 3.10) as model compounds. The present study was inspired by the demand for more environmentally friendly procedures and the need to reduce sample preparation time. Table 1 depicts the chemical structures and properties of the pharmaceutical compounds studied.

**Table 1:** Pertinent information on the pharmaceutical compounds studied^a^

Compounds	Structure	Log *P*	p*K*_a_
Metformin (MET)	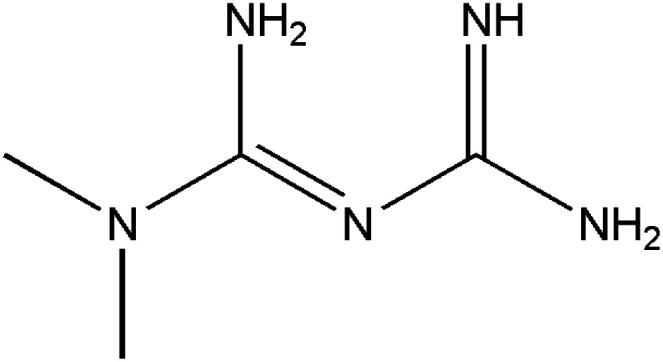	−1.82^a^	11.5^b^
Buformin (BUF)	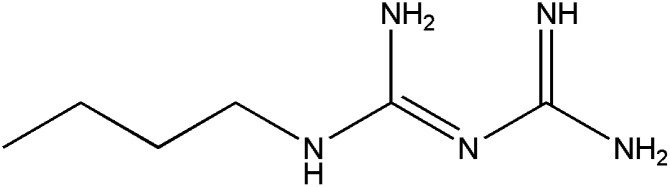	−1.20^c^	12.27^c^
Phenformin (PHEN)	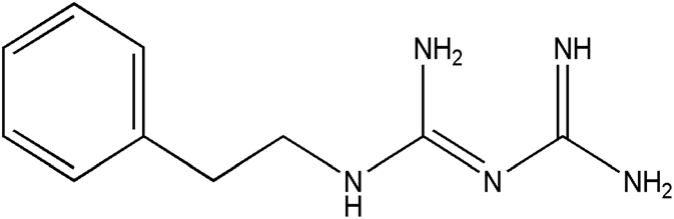	0.41^a^	11.8^e^
Propranolol (PROP)	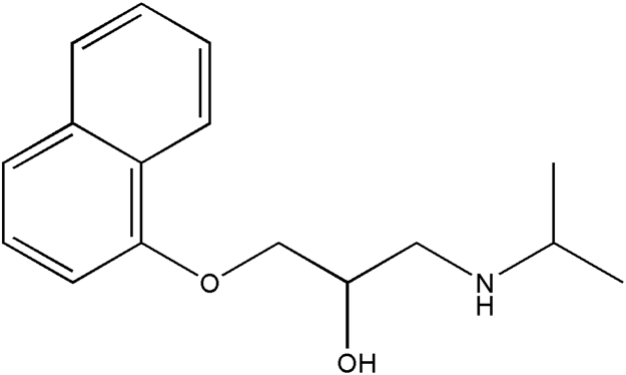	3.10^a^	9.49^f^

a a Calculated using Chemsketch from ACDlabs; b, d–f ref. 37–40; c SciFin.

## 2.0 Experimental

### 2.1 Chemicals and reagents

Chemicals and reagents used were procured from the following sources: graphene nanoplatelet, metformin hydrochloride (97%), phenformin hydrochloride (97%), propranolol hydrochloride (99%), triethylamine, sodium monophosphate were purchased from Sigma Aldrich (Steinheim, Germany); zeolite Linde Type L (LTL) (Tosoh Corporation, Japan); buformin hydrochloride (95%) was from Wako Pure Chemicals Industries (Osaka, Japan); HPLC grade methanol (MeOH) and acetonitrile (ACN), and sodium chloride were from Quality Reagent Chemicals (QReC, Auckland, New Zealand). HPLC grade tetrahydrofuran (THF) (>99.9%), 2-propanol (IPA), dichloromethane (DCM) sodium hydroxide, orthophosphoric acid were obtained from Merck (Darmstadt, Germany). Toluene and acetic acid (99.8%) were purchased from HmbG Chemicals (Hamburg, Germany). Ultrapure water (resistivity, 18.2 MΩ cm^−1^) was produced from Millipore water (Molsheim, France) purification system and was used throughout. Polypropylene (PP) sheet membrane (Accurel 2E HF (R/P), 166 μm thickness, 0.2 μm pore size) was purchased from Membrana (Wuppertal, Germany).

### 2.2 Preparation of bar-μ-SPE device

**Fig. 1:**
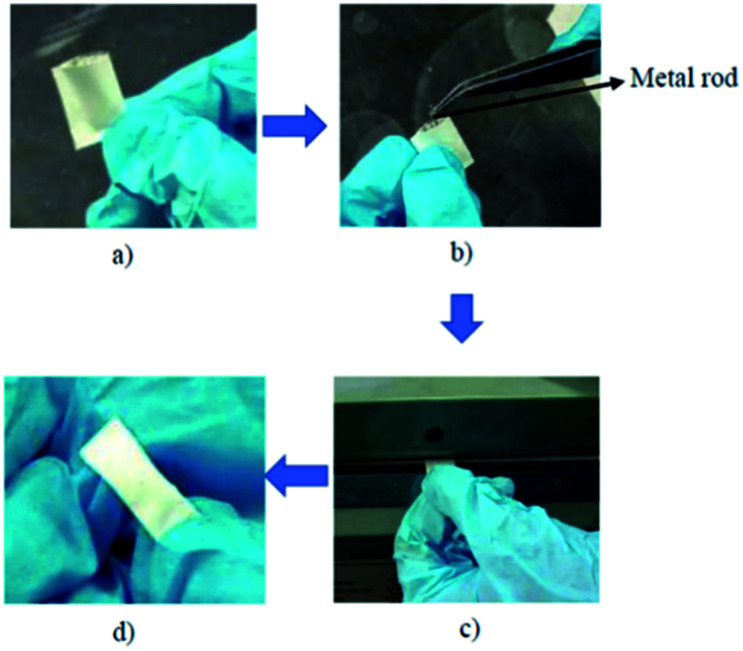
Preparation of bar-μ-SPE. (a) Preparation of PP bag (b) insertion of metal rod in PP bag (c) heat-sealed of edges (d) completed device.

The bar-μ-SPE extraction device was set up (Fig. 1) according to the approach adopted by Alshishani and co-workers.^25^ First, the polypropylene (PP) membrane was cut (2.4 × 1.8 cm), folded into half and heat-sealed on both sides. The adsorbent (20 mg) was then inserted through the open edge. Next, a tiny steel metal rod (diameter, 1 mm; length, 1.1 cm) was also added and the open edge was completely heat-sealed. The metal rod was acting like a magnetic bar itself. The PP membrane bag was eventually folded into half and heat-sealed again. The size of the prepared bag was 1.5 × 0.4 cm. The device was soaked in acetonitrile and sonicated for 5 min before use.

### 2.3 Instrumentation

Alliance model 2695 HPLC unit was obtained from Waters (Milford, MA, USA). The instrument was equipped with a photodiode array detector (DAD model number 2998) was set at 230 nm. The separation was carried out using an Agilent Zorbax TMS C1 column (5 μm, 80 Å, 4.6 × 250 mm). The targeted compounds were separated by a mixture of acetonitrile : phosphate buffer (pH 6.2, containing 20 mM of sodium monophosphate) : triethylamine (45 : 55 : 0.2, v/v). The mobile phase was filtered through a Nylon membrane filter (0.22 μm, Agilent Technologies, Waldbronn, Germany). It was also freshly prepared and degassed for 15 min before use. Isocratic elution at a flow rate of 1.3 mL min^−1^ with a volume of injection of 20 μL was employed for the analysis. The data were processed using licensed Empower V.2 software (Milford, MA, USA). The log *P* and p*K*_a_ value for each drug were calculated using Chemsketch from ACDlabs.

### 2.4 Preparation of standard solutions

MET, BUF, PHEN, and PROP stock solutions (500 mg L^−1^) were prepared by dissolving appropriate amounts in deionized water, where the stock solutions were stored in a refrigerator at 4 °C until further usage. Working solutions were prepared from the stock solutions by diluting with an appropriate volume of deionized water.

### 2.5 Urine samples

Human urine samples were obtained from volunteers of healthy postgraduate students from the School of Chemical Sciences, Universiti Sains Malaysia in February 2019. The criteria for sample collection are the student do not take any medications containing those targeted analytes. Urine samples were spiked with the desired concentration of the targeted analytes and diluted with deionized water (1 : 4, v/v). The urines samples were stored in a refrigerator at 4 °C and used within 2 days.

### 2.6 Extraction procedure

The bar-μ-SPE device was cleaned by putting it in acetonitrile for 7 min. The device was then immersed in a sample solution (20 mL), which was stirred at 800 rpm for 60 min to carry out the extraction process. Next, the device was removed from the sample solution, washed with deionized water and dried using lint-free tissue paper. The device was inserted into a centrifuge tube containing acetonitrile as a back-extraction solvent. The desorption process was accomplished by sonicating the bar-μ-SPE device for 30 min. Finally, 20 μL of the extract was directly injected into the HPLC unit.

### 2.7 Extraction optimization

Optimization parameters of the chosen adsorbents were performed in triplicates, and the mean value was used for high accuracy. The percentage of extraction (% *E*) and enrichment factor (EF) was used to choose the optimum amount. EF was determined by comparing the area of each analyte in aqueous standard (1000 μg L^−1^) subjected to the extraction procedure, to the area before extraction.1
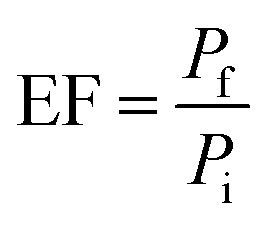
where *P*_f_ is the final peak area in desorption solvent and *P*_i_ refers to the initial peak area in the aqueous sample.

## 3.0 Results and discussion

### 3.1 Optimization of HPLC conditions

The wide range of polarity of targeted drugs makes it challenging to find suitable reverse-phase chromatographic conditions.^3,41,42^ Rapid elution of biguanide compounds, in particular MET on C18 column resulting in interference with the solvent occurred due to their hydrophilicity.^43^ This issue can be addressed by adding ion pair additives to the mobile phase, such as sodium dodecyl sulphate or sodium heptane sulfonate.^44^ The presence of an optimum concentration of ion-pair forming additive reduces its polarity.^43^ A derivatization agent, *p*-nitrobenzoyl chloride was used to derivatize MET into a less polar compound.^45^ According to a recent study, the C1 column provided an excellent separation without using any derivatization agent or ion pair. This is attributable to the higher polarity of C1 column compared to C18, C8, and pentafluoro phenyl (PFP) columns.^46^

Mixtures of acetonitrile with phosphate buffer at different compositions as the mobile phase were investigated on the C1 column. The targeted drugs were well separated with 40–60% of acetonitrile was used. The retention times of MET, BUF and PHEN increased as acetonitrile composition increased, while PROP demonstrated a contrasting behaviour as it is the most hydrophobic drug. The retention time of PROP decreased with the increasing acetonitrile composition. Phosphate buffer (pH 6.2) containing 20 mM sodium monophosphate, was found to be a promising component of the mobile phase, together with acetonitrile (ACN). The lower pH of the buffer shortens the retention time of analytes. In view of improving the separation, a small amount of triethylamine was added to the mobile phase to reduce the peak tailing. Several mobile phase compositions were tested, with the optimum was found to be 45 : 55 : 0.2 (phosphate buffer : ACN : triethylamine, v/v). Substituting acetonitrile with methanol resulted in an overlapping peak for biguanide compounds. Methanol has a polarity index of 6.6, which is higher than acetonitrile (6.2).^45^ A polarity index of 6.6 indicates that methanol is more polar than acetonitrile. Therefore, the higher the polarity of the mobile phase with methanol is used, which induced the overlapping of hydrophilic biguanide peaks. All analytes were separated and eluted within 13 min. The order of elution is following their lipophilicities.

### 3.2 Type of adsorbent

The success of the bar-μ-SPE predominantly lies in the choice of a suitable adsorbent. The retention of an analyte by an adsorbent is governed mainly by weak interactions, such as hydrogen bonding, π–π, electrostatic and van der Waals interactions. This facilitates the desorption and the possible regeneration of the adsorbent for repeated use. Thus, adsorbents (zeolite and graphene) were tested by placing 20 mg of the particular adsorbent into the device. Adsorbents of different polarities were chosen as the targeted analytes to encompass a wide range of polarities. Initially, each adsorbent was tested one at a time by extracting 1 mg L^−1^ of the drugs' mixture. The concentration of the remained drugs in the solution was analyzed using HPLC. Under these unoptimized conditions, the % *E* for MET, BUF, PHEN and PROP using graphene were 17.9, 17.4, 20.9 and 33.5%, respectively, while using zeolite were 14.6 (MET), 15.4 (BUF), 14.6 (PHEN) and 30.4% (PROP).

Due to its desirable particle size of 4 μm, the zeolite adsorbent was easy to handle. The use of graphene involves a special introduction into the bag, was accomplished using a modified filter funnel fitted with a tip at the end. Besides, graphene sticking to the wall of the PP bag created difficulties in the heat-sealing process. As a result, the PP bag was folded into half to achieve constant rotation. Critical parameters affecting the extraction processes were systematically investigated.

### 3.3 Optimization of bar-μ-SPE procedure

Several parameters were tested to evaluate diverse factors affecting the extraction capability of each drug. The optimization was carried out by analyzing in triplicate using the drug mixtures (1 mg L^−1^). The parameters studied were the type of conditioning solvent, amount of adsorbent, pH of the sample, the effect of salt addition, extraction time and extraction speed. The conditions for desorption investigated were solvent type, the volume of solvent and desorption time.

#### 3.3.1 Type of conditioning solvent

Prior to the extraction process, the bar-μ-SPE device should be immersed in an organic solvent to activate the membrane for better analyte diffusion, as well as conditioning the adsorbent and cleanse it from contaminants. Thus, several commonly used pre-conditioning solvents, such as 3% acetic acid, MeOH, ACN, THF and toluene were tested. The best pre-conditioning solvent was ACN for both adsorbents. Therefore, ACN was used for further assays.

#### 3.3.2 Effect of pH

The pH of the sample solution significantly affects the extraction, particularly for amines and other ionizable compounds. Varying the pH changes the existing form of analytes in solution. Thus, the effect of solution pH (3–10) on the extraction of the drugs were studied.

**Fig. 2:**
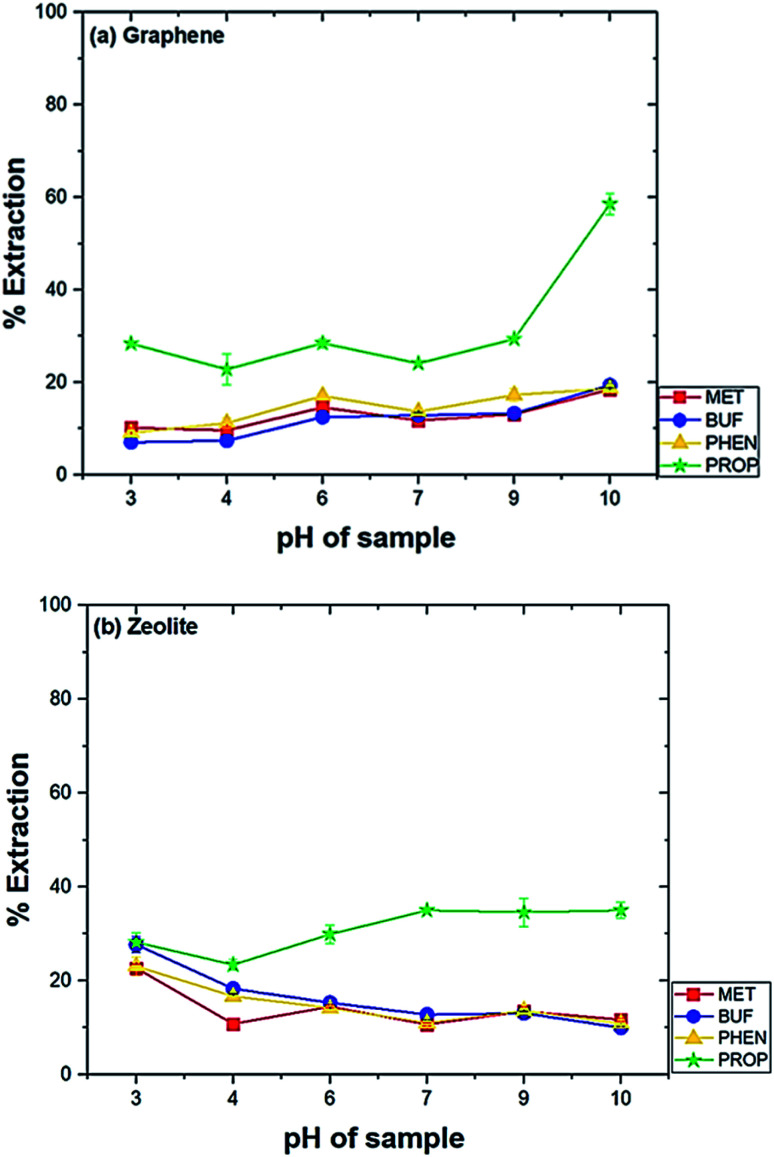
Effect of pH on the extraction of drugs. Experimental conditions: conditioning solvent, ACN; volume of extraction solvent, 20 mL; amount of adsorbent, 20 mg; time of extraction, 60 min; rotation speed, 800 rpm.

The highest extraction was obtained at pH 10 when graphene was used (Fig. 2(a)). The extraction was preferred under basic condition can be explained by the favourable adsorption of the neutral form of the drugs. Meanwhile, at lower pH, the amine group will be protonated. This phenomenon leads to the low extraction efficiency of biguanide compounds since the membrane is hydrophobic. For zeolite, the highest extraction efficiency for biguanide compounds occurred at pH 3 (Fig. 2(b)).

This is because when in acidic media, the drugs get protonated and become positively charged. Therefore, low pH promotes the adsorption of the analytes on to zeolite due to the predominant negative charges on zeolite. This effect has also been observed in an earlier study. In a previous study using zeolite LTL to extract ochratoxin A in coffee and cereal under acidic condition in order to deionize the molecule and to promote its extraction (pH 1.5–3.0). The study discovered the optimum pH was at pH 1.5.^36^ Hence, the sample solution at pH 10 was used for graphene and pH 3 for zeolite in further studies.

#### 3.3.3 Amount of adsorbent

The effect of amount of adsorbents on the extraction of the drugs were studied from 10 to 25 mg. The optimum results were obtained when 10 mg of graphene and 25 mg of zeolite were used. The smaller mass of graphene was suitable due to its large surface area (750 m^2^ g^−1^) compared to zeolite (290 m^2^ g^−1^). In order to accommodate more than 20 mg of graphene, a larger PP bag with dimension >1.5 × 0.4 cm should be used.

#### 3.3.4 Effect of extraction time and stirring speed

**Fig. 3:**
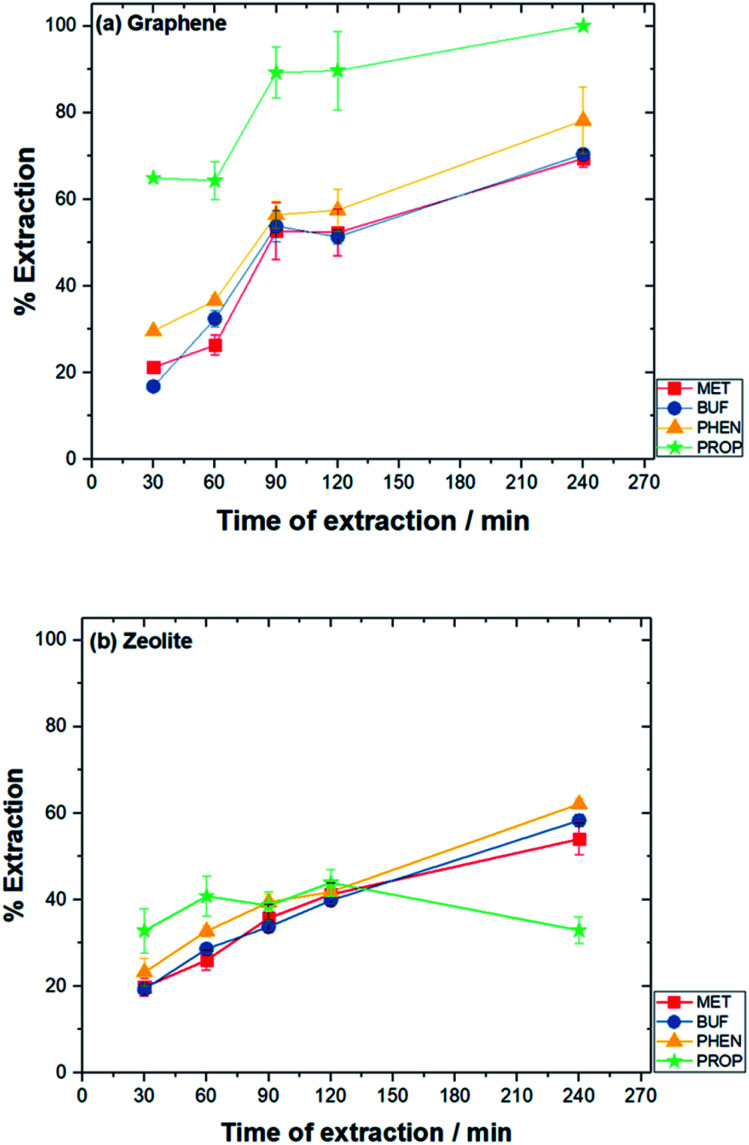
Effect of time of extraction on the extraction of drugs. Experimental conditions: conditioning solvent, ACN; volume of solution, 10 mL; pH solution, pH 10 (graphene) and pH 3 (zeolite); amount of adsorbent, 10 mg (graphene) and 25 mg (zeolite); rotating speed, 800 rpm.

It is vital to examine the time of the extraction for determining the time needed for the compounds to migrate from the solution towards the adsorbent. The effect of extraction time (30–240 min) of the drug mixtures were analyzed. The efficiency increased with time until about 90 min when graphene was used as compared to 120 min for zeolite (Fig. 3). For subsequent tests, 90 and 120 min were selected for graphene and zeolite, respectively.

Stirring of the solution enhances the mass transfer of analytes toward the adsorbent materials. The bar-device itself served as a magnetic bar. The effect of different stirring rates from 600 to 1200 rpm of the drug mixtures was evaluated on the extraction. Stirring at 800 rpm was found to be the optimum for both adsorbents (results not shown).

#### 3.3.5 Effect of salt (NaCl) addition

The influence of ionic strength was examined by the adding about 0–10% of sodium chloride (NaCl) to 10 mL of sample solution during the extraction process. The addition of salt results in decreased solubility of analytes in the sample solution and facilitates their migration to an adsorbent or so-called ‘salting-out’ effect.^47^ It seems that the addition of salt did not improve the % *E*. Hence, further assays were continued without the addition of salt for both adsorbents.

#### 3.3.6 Effect of desorption solvent, time and volume

Upon extraction, the analytes were desorbed with the aid of ultrasonication after adding a suitable organic solvent. The added organic solvent must be able to disrupt the interaction between analyte and adsorbent.

High desorption was obtained when an ion-pair reagent (IPA) was used for graphene adsorbent. To further enhancing the desorption process, IPA (0.1 M sodium heptanesulphonate) was added to the solvent. A significant increase in peak area for MET and BUF was observed. ACN was found to be the most suitable desorption solvent for zeolite. Moreover, the desorption significantly increased for MET, BUF and PHEN with 0.1 M sodium heptanesulphonate was added to the ACN–water mixture. Pertaining to graphene (non-polar adsorbent), BUF (polar analyte) recorded the highest enrichment factor, whereas, for zeolite (polar adsorbent), it was PROP (non-polar analyte). The incompatibility between adsorbent and analyte resulted in the weak interaction. Hence, it was affirmed that it is easier to disrupt the interaction and back-extract them into the desorption solvent.

The analytes were desorbed ultrasonically using suitable solvents. Different sonication times were established (15–60 min) with the maximum peak area after desorption was obtained with adopted 30 min of desorption time for both adsorbents. After 30 min, the analytes could degrade.^48^ The other potential explanation is that the analytes might get re-adsorbed by the adsorbent material.^49^ Different volumes of desorption solvent were examined, *i.e.*, 0.6, 0.8 and 1.0 mL. The highest peak area was found with 0.6 mL of desorption solvent was used for both adsorbents.

#### 3.3.7 Optimized conditions for single adsorbent

The optimized conditions are summarized in Table 2. Under these conditions, the extraction efficiency, the % EE obtained using graphene were: MET (13.9%), BUF (15.3%), PHEN (5.03%) and PROP (9.13%). The % EE was calculated from the peak area obtained from the HPLC chromatogram. The equation is depicted as below:2
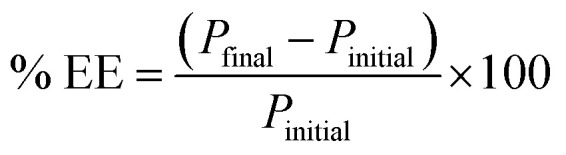


**Table 2:** Summary of the adopted conditions of bar-μ-SPE-HPLC method using graphene and zeolite as adsorbent^a^

Conditions	Graphene	Zeolite	Zeolite : graphene
Conditioning solvent	ACN	ACN	ACN
pH of sample solution	10	3	6
Volume of sample solution, mL	10	10	10
Amount of adsorbent	10 mg	25 mg	24.5 mg : 10.5 mg
Rotating speed, rpm	800	800	800
Time of extraction, min	90	120	120
Ionic strength, % NaCl	0	0	0
Solvent for desorption	0.1 M IP in IPA	0.1 M IP in ACN	7 : 3 (ACN : IPA) + 0.1 M IP
Time of desorption, min	30	30	30
Volume of desorption solvent, mL	0.6	0.6	0.6

a IP: ion-pair (sodium heptanesulphonate).

With regard to zeolite, % EE were: MET (7.57%), BUF (17.0%), PHEN (28.8%) and PROP (39.2%). Under the aforementioned optimum conditions, the EF for graphene were: 4.42, 4.76, 1.49 and 1.71 for MET, BUF, PHEN, and PROP, respectively, while for zeolite were: 3.07, 7.12, 11.5 and 14.9, respectively.

### 3.4 Effect of mixed-adsorbent

Mixtures of graphene and zeolite as adsorbents were employed to enhance the extraction efficiency of not well extracted drugs using a single adsorbent (*e.g.* PHEN and PROP when graphene was used and MET when zeolite was used for extraction). Investigation of the influence of mixed-adsorbents was initially carried out based on the optimized conditions adopted for individual adsorbents. The time of extraction was 120 min.

Different zeolite : graphene (w : w) composition as adsorbents were studied, *i.e.*, 30, 50 and 70%. Mixed-adsorbents were prepared by adding zeolite first into the extraction device, followed by the addition of graphene. The optimum results were obtained with 70% zeolite was used, which was used for further assays.

**Fig. 4:**
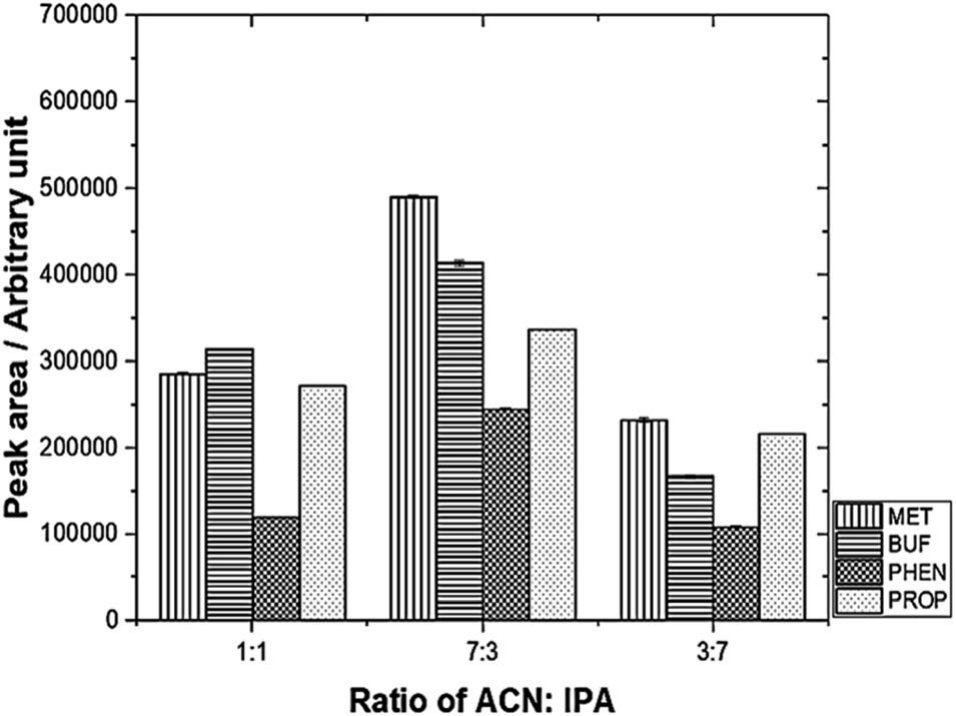
Effect of different desorption solvent mixtures on peak area using mixed adsorbent. Experimental conditions: volume of desorption solvent, 0.60 mL; sonication time, 30 min.

The desorption solvent using a mixture of 0.1 M IPA and ACN was investigated. The results are depicted in Fig. 4. The optimum volume ratio (v/v) for ACN : IPA was found to be 7 : 3.

The other parameters, such as pH and desorption, were also studied, and the final adopted conditions are summarized in Table 2. These conditions were employed for further studies.

**Fig. 5:**
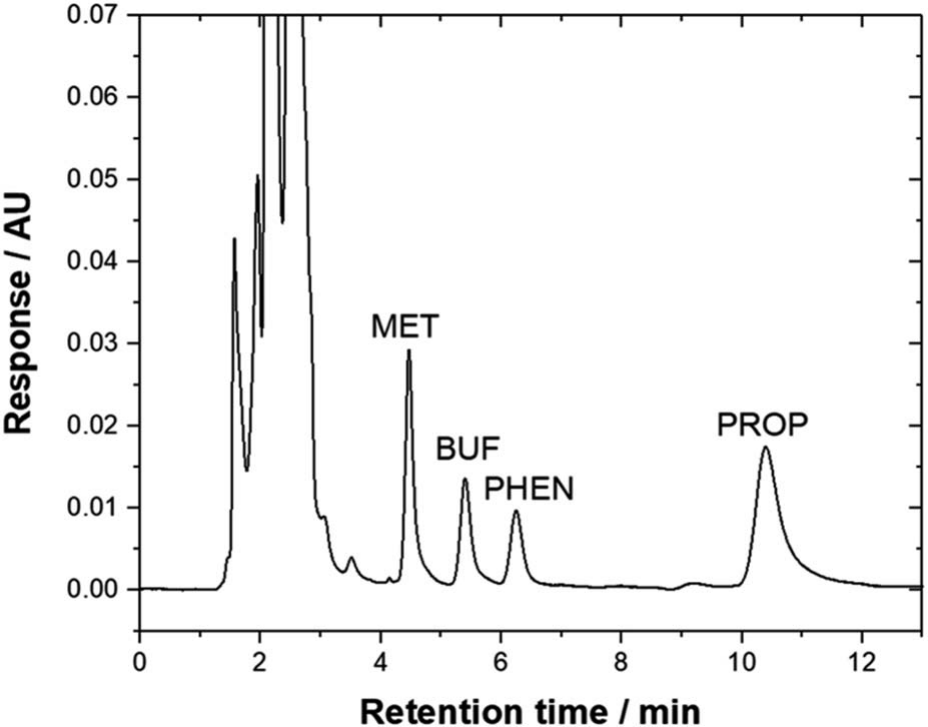
Typical chromatogram of urine sample that was spiked with 950 μg L^−1^ drugs and subjected to the bar-μ-SPE-HPLC method using mixed-adsorbent. Chromatographic conditions: column, Zorbax TMS (250 × 4.6 mm); mobile phase, 20 mM phosphate buffer (pH 6.2) : ACN : triethylamine (45 : 55 : 0.2, v/v); *λ*, 230 nm; flow rate, 1.3 mL min^−1^.

Method validation was performed by spiking drug-free urine samples (blank analysis was performed before use). Linearity was studied using seven concentrations of standard mixtures (17–1000 μg L^−1^). The calibration curve was established by plotting the peak area *versus* concentration of each targeted compounds. The calibration curve was linear with a high regression coefficient over the concentration range studied. All the analytes demonstrated to be well-correlated (*R*^2^ > 0.99). The limit of detection (LOD) and limit of quantification (LOQ) were calculated based on the signal-to-noise ratio (S/N) of 3 and 10 as the background signal noise, respectively. The ratio between peak intensity and intensity of the noise was used^50^ and the value obtained was 3.94–17.6 μg L^−1^. Precision, expressed as RSD was measured using three different concentration levels (600, 750 and 950 μg L^−1^) for all compounds. Excellent repeatability for the intra-day and inter-day were obtained (% RSD < 9.12, Table 3). Recovery studies were carried out by spiking three concentrations of standard mixtures into the urine sample (600, 750, and 950 μg L^−1^). Table 3 shows good recoveries were obtained for all targeted analytes. Fig. 5 illustrates a typical chromatogram of a urine sample subjected to the bar-μ-SPE method. The analytes were well separated from the matrix components.

**Table 3:** Interday and intraday reproducibility (% RSD) as well as recovery for urine spiked sample (*n* = 6) using mixed-adsorbent

Parameter, spiked level (μg L^−1^)	MET	BUF	PHEN	PROP
**Intraday (% RSD, *n* = 6)**				
600	6.66	6.91	7.35	8.40
750	5.16	8.28	8.62	6.90
950	4.99	6.27	5.73	5.87

**Interday (% RSD, *n* = 6)**				
600	8.28	8.62	8.55	9.12
750	8.02	8.87	9.01	7.80
950	6.50	7.23	7.41	7.67

**Recovery (%)**				
600	85.4 ± 8.98	84.6 ± 10.2	109 ± 8.15	97.0 ± 8.58
750	75.1 ± 6.92	72.8 ± 8.53	114 ± 13.5	116 ± 16.7
950	80.9 ± 4.57	81.2 ± 1.76	116 ± 8.95	97.8 ± 7.06

### 3.5 Comparison of performance between individual and mixed-adsorbents

**Fig. 6:**
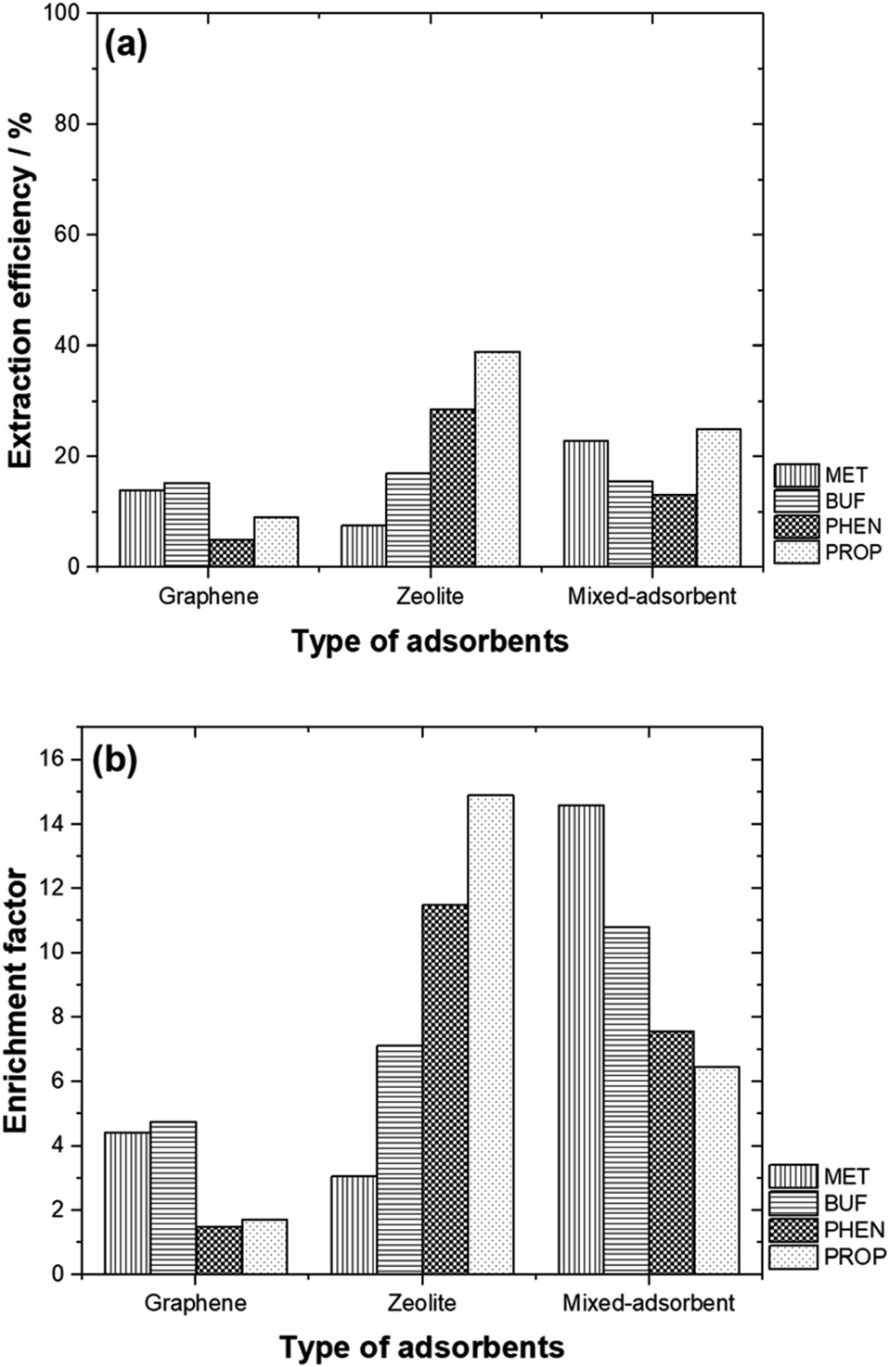
(a) EE % and (b) EF values for different types of adsorbents studied.

Comparison of the % EE using the adsorbents studied is shown in Fig. 6(a). Generally, zeolite and the adsorbent mixture offered better % EE compared to graphene. It is also interesting to note that, PHEN and PROP (non-polar compounds) are well extracted with higher EF as zeolite was used as an adsorbent. In contrast, MET and BUF (polar compounds) demonstrated high % EE and EF using mixed-adsorbent. The hydrophobicity of these compounds allows them to be desorbed easily than polar compounds. In general, the EF obtained were typical of adsorbents based on SPE. Pertaining to zeolite, high adsorption capability toward polar molecules reflected their strong electrostatic and guest–host interactions with the micropore channels of zeolite. The targeted compounds were preferably adsorbed on to the graphene due to the π–π interaction and their hydrophobic properties.

By mixing both adsorbents, the % EE only increased for MET. The highest % EE was obtained for BUF, PHEN and PROP with zeolite was used. Fig. 6(b) compares the EF between the different adsorbents studied. The highest EF values were obtained for polar compounds (MET and BUF) with mixed-adsorbents were used. Meanwhile, zeolite offered the highest EF for PHEN and PROP.

### 3.6 Comparison with previously reported analytical methods

The analytical characteristics of the proposed bar μ-SPE-HPLC method was compared with some previously reported methods, as shown in Table 4. The table showed that SPE is the most frequently adopted sample preparation method. The proposed method is simple, and unlike conventional SPE method, it does not require a lengthy evaporation step.^51–53^ The tested drugs demonstrated good recoveries when spiked to urine samples (72.8–116%). Another strength of the work lies in the fact that the high potential of the extracting the compounds with diverse polarities simultaneously. It is noteworthy that current methods for the analysis of these compounds required different extraction and separation conditions, thus making the investigation time consuming and more expensive.^54^

**Table 4:** Comparison of previously reported methods for the simultaneous determination of analytes^a^

Sample preparation and determination	Sample	Analytes	p*K*_a_	Log *P*	LOD (μg L^−1^)	LOQ (μg L^−1^)	Recovery (%)	Ref.
IP SPE-IP LC	Plasma	MET	—	—	3	5	97.9–100.5	51
SPE-non-aqueous CE	Plasma	MET	131	—	12	—	—	54
		PHEN	12.7		6	—	—	
IP SPE-HPLC MS	Plasma	MET	—	−1.80	—	2.49	86.1	55
Bar-μ-SPE-HPLC UV	Urine	MET	11.5	−1.82	4.03	12.2	75.1–85.4	This work
		BUF	12.3	−0.03	3.94	11.9	72.8–84.6	
		PHEN	11.8	0.41	6.90	20.9	109–116	
SPE-LC ESI/MS MS	Plasma	PROP	—	—	0.05	0.20	96.4–98.5	52
		4-HYD PROP	—	—	0.10	0.20	64.7–66.2	
EME-GC MC	Wastewater	PROP	—	—	0.0081	—	80.0	14
		NOR	—	—	0.13	—	18.0	
		IBU	—	—	0.13	—	20.0	
		ALP	—	—	0.18	—	23.0	
		NAP	—	—	0.26	—	26.0	
		KETO	4.2–9.5	—	0.027	—	40.0	
MIP SBSE-HPLC UV	Urine	PROP	9.50	2.90	0.37	1.0	86.8–106	56
SPE-UHPLC UV	Urine	PROP	—	—	19	59	85.5–105	53
Bar-μ-SPE-HPLC UV	Urine	PROP	9.49	3.10	17.0	51.6	97.0–116	This work

a Abbreviations: MISPE: molecularly imprinted solid phase extraction, MIP: molecularly imprinted polymer, TFA: trifluoroacetic acid, IP SPE: ion pair solid phase extraction, IP LC: ion pair liquid chromatography, 4-HYD PROP: 4-hydroxy propranolol, EME: electromembrane extraction, NOR: norephedrine, IBU: ibuprofen, ALP: alprenolol, NAP: naproxen, KETO: ketoprofen, MIP SBSE: molecularly imprinted polymer stir-bar sorptive extraction, UHPLC: ultra high-performance liquid chromatography, ESI: electrospray ionization, MS: mass spectrometry.

## 4.0 Conclusions

An alternative method for the extraction of compounds of diverse polarity using mixed-adsorbents of graphene and zeolite in the bar μ-SPE format was demonstrated using MET, BUF, PHEN and PROP as model compounds. The proposed approach capitalizes on the favourable characteristics of the bar μ-SPE technique, such as simplicity, conservation of materials and minimization of interferences from macromolecules provided by the membrane. Through the sensible choice of adsorbents, the method can be tailored for the extraction of compounds of interest. The extracts are compatible with HPLC method, thus can be directly analyzed. The use of a slightly polar C1 HPLC column demonstrated its potential to overcome the inconvenience of working with IP reagent as compared to C18 column in the separation of polar drugs, such as MET. Baseline separation of the peaks was obtained in about 11 min.

## Live subject statement

All experiments were performed in accordance with the guidelines of urine sample collection and Universiti Sains Malaysia do not require Ethics Committee approval to use urine from student volunteers, however the students gave consent to the use and voluntarily collect the samples. Ethics only required when blood are withdrawn, research involving human tissue and DNA analysis.

## Author contributions

Maizatul Najwa Jajuli: methodology, validation, formal analysis, investigation, writing – original draft, writing – review and editing. Grégoire Herzog: writing – review and editing, resources. Marc Hébrant: writing – review and editing, resources. Ng Eng Poh: resources. Afidah Abdul Rahim: writing – review and editing, resources, supervision. Bahruddin Saad: conceptualization, methodology, writing – original draft, resources, supervision. M. Hazwan Hussin: writing – review and editing, resources, supervision, project administration, funding acquisition.

## Conflicts of interest

The authors declare no conflict of interest.

## Supplementary Material
